# Late Removal of Titanium Hardware from the Elbow Is Problematic

**DOI:** 10.5402/2012/256239

**Published:** 2012-02-06

**Authors:** Abdo Bachoura, Ruriko Yoshida, Christian Lattermann, Srinath Kamineni

**Affiliations:** Elbow Shoulder Research Center, Department of Orthopaedic Surgery and Sports Medicine, University of Kentucky, 740 South Limestone Street, K-412 Kentucky Clinic, Lexington, KY 40536-0284, USA

## Abstract

A retrospective review of 21 patients that underwent bone screw removal from the elbow was studied in relation to the type of metal, duration of implantation, and the location of the screws about the elbow. Screw failure during extraction was the dependent variable. Five of 21 patients experienced hardware failure during extraction. Fourteen patients had titanium alloy implants. In four cases, titanium screws broke during extraction. Compared to stainless steel, titanium screw failure during removal was not statistically significant (*P* = 0.61). Screw removal 12 months after surgery was more likely to result in broken, retained screws in general (*P* = 0.046) and specifically for titanium alloy (*P* = 0.003). Bone screws removed from the distal humerus or proximal ulna had an equal chance of fracturing (*P* = 0.28). There appears to be a time-related association of titanium alloy bone screw failure during hardware removal cases from the elbow. This may be explained by titanium's properties and osseointegration.

## 1. Introduction

**Figure 1:**
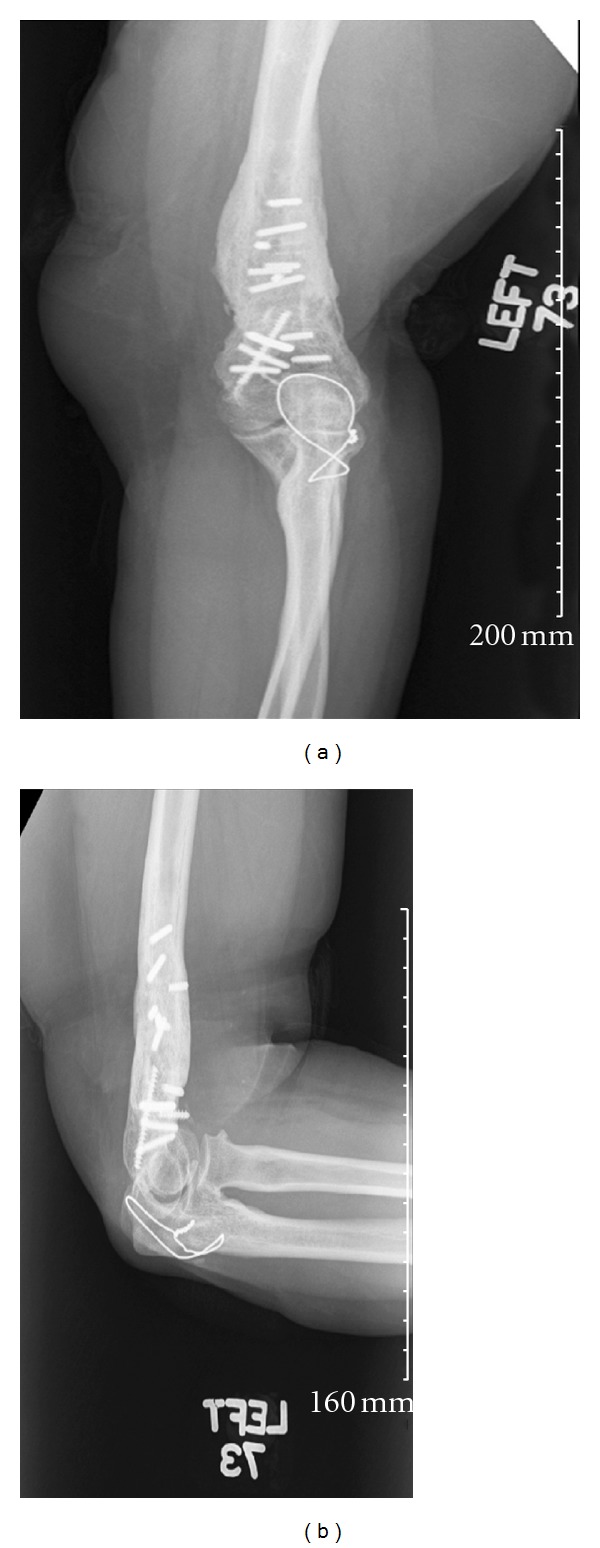
Broken hardware often poses a difficult problem and subsequent removal may increase surgical time and complexity of future surgeries. (a) shows an X-ray of an elbow (AP view) with broken and retained screws. (b) shows the same elbow in the lateral position.

Hardware removal is indicated for infection, nonunion, failure of fixation, pain, soft tissue irritation, and anticipated strenuous activity after fracture healing [1–4]. During removal cases however, hardware, especially screws, can break. Subsequent removal of broken hardware increases surgical time, and retained metalwork potentially complicates future surgeries (Figure 1) [5]. 

Although there have been several articles that have discussed titanium implant failure, most have discussed this issue within the context of hardware failure during fracture healing, and not particularly during removal of hardware [6–9]. To our knowledge, none have been specific to the elbow, which merits its own discussion due to its unique anatomy. The distal humerus of the elbow is unique in that is has a high ratio of cortical to cancellous bone. Therefore, in this study we set out to investigate incidence of bone screw failure during hardware removal procedures and we were interested in comparing titanium and stainless steel bone screws because these are the most common types of metallic fracture implants in circulation. In addition, we set out to determine whether the duration of implantation and the anatomic location of the bone screws about the elbow were associated with bone screw failure during removal procedures. A better understanding of metallic hardware failure during removal procedures may help surgeons in the preoperative planning stages of these cases, in terms of surgical tool selection and staff availability.

## 2. Methods

After Institutional Review Board (IRB) approval, all cases performed by orthopaedic trauma or upper extremity surgeons between 1/1/2000 and 10/1/2009 at our level 1 trauma center were reviewed. Inclusion criteria were (1) deep implant removal cases, (2) hardware removed from the distal humerus or the proximal ulna, and (3) isolated elbow injuries. The exclusion criteria were (1) cases that did not have relevant or inaccessible elbow X-rays, (2) single screw fragment extraction cases (because in these cases the hardware had previously broke and was small in size, which we believe was not representative of the other screws being removed), (3) patients younger than 17 years, and (4) cases that were originally performed at an outside institution (unavailable medical records). The factors considered were (1) whether or not the bone screws broke during removal and the type of implant metal used (Titanium alloy, Ti6Al4V or Stainless Steel), (2) the length of time between initial implantation and removal, where cases were divided into two groups based on a conservative estimate of the time period required for osseointegration of titanium implants [10, 11]: one group was for cases where the duration of time between implantation and removal was less than 12 months and the second group was for cases where the duration between implantation and removal was 12 months or more; and (3) anatomic location about the elbow (distal humerus or proximal ulna). The data was extracted from the medical record.

Due to the small sample size, Fisher's exact test was used to determine statistical differences between two sets of categorical data. An independent *t*-test was used to compare the means of two independent groups. Differences that had less than 0.05 probability of occurring from chance were considered statistically significant.

## 3. Results

We identified a total of 47 cases, of which 21 met the inclusion criteria. The mean age of patients was 38.7 (17–66) years. We carried out an independent *t*-test to determine if there were any differences between the ages of patients that had broken screws and those that did not, and no statistical significance was found, *P* = 0.740. Out of 21 cases, screws broke during removal in 5 cases (23.8%). In 16 out of 21 cases, hardware was removed without breaking. The reasons for hardware removal were infection in 7/21 cases, symptomatic, prominent hardware in 7/21 cases, nonunion in 6/21 cases, and contracture in 1/21 cases.  Table 1 lists a summary of our findings.

Out of 21 cases, 14 involved titanium alloy and 7 involved stainless steel implants. Within the titanium hardware group, in 10 cases removal was uneventful, and in 4 cases, fracture of at least one screw occurred. In comparison, out of the 7 stainless steel hardware removal cases, there was one case that resulted in one or more broken screws. Overall, compared to stainless steel, failure of titanium alloy screws during removal was not found to be statistically significant (*P* = 0.61). 

In order to determine whether there were any association between the duration of implantation and hardware failure during removal, cases were divided into two groups: Group (1) duration of hardware implantation was 12 months or less (mean 7.7, range two to 12 months), and Group (2): duration of implantation was more than 12 months (mean 41.6, range 16 to 74 months). Twelve cases had hardware removed within 12 months of implantation and nine cases had hardware removed after 12 months of initial implantation. Bone screws that were removed after 12 months of surgery were more likely to break during removal (*P* = 0.046). When titanium screws were analyzed separately, those removed within 12 months of surgery were more likely to be removed intact as compared to those removed more than 12 months after implantation (*P* = 0.003). The small number of stainless steel cases (seven) did not warrant statistical calculations.

With respect to anatomic location, there were 12 distal humerus and 15 proximal ulna cases (Table 1). Six cases involved the distal humerus only, nine cases involved the proximal ulna only, and six cases had simultaneous proximal ulna and distal humerus involvement. In one case where titanium screws broke and in one case where stainless steel screws broke, it was unclear where the location was and these cases were discarded from the analysis. In general, bone screw failure was equally likely to occur when removed from the distal humerus and the proximal ulna (*P* = 0.28).

## 4. Discussion

Hardware failure during removal cases is a commonly seen problem in orthopaedics [5] (Figure 1). Currently, there is no single hardware removal technique that is uniformly successful, and several different methods may be employed during the same case. Such techniques include the use of screw extractors, trephines, extraction bolts, pliers, and various other devices [5]. The purpose of this article was to determine the incidence of bone screw failure during hardware removal procedures, and we were interested in comparing titanium and stainless steel bone screws. In addition, we set out to determine whether the duration of implantation and the anatomic location of the bone screws about the elbow had any association with bone screw failure during removal procedures.

We believe that prior knowledge of the type of metal implanted (mainly Titanium) and the duration of implantation to be useful information that can help in the preoperative planning of hardware removal procedures. Firstly, this may allow surgeons to request hardware removal kits, thus saving precious operative time. Second, it is our experience that hardware removal procedures are often considered not technically demanding and are often delegated to less experienced surgical staff such as junior residents who may be more likely to break the hardware. Therefore, we believe that experienced staff surgeons should be available during procedures where titanium is being removed. Having broken hardware in the elbow may complicate future surgeries in the same region of the limb.

With regards to orthopaedic implants, it is known that both titanium alloy and commercially pure titanium hardware are more predisposed to in situ fracture relative to stainless steel [6–9]. As compared to stainless steel, titanium alloy is lighter, has a lower modulus of elasticity, and has superior corrosion resistance and biocompatibility, but inferior ductility and notch sensitivity. The literature search performed for this review did not reveal any previous studies that compare hardware removal from the elbow in vivo for titanium and stainless steel fracture implants.

In contrast to titanium implants remaining in situ for less than 12 months, we observed that the titanium implants remaining in situ for more than 12 months had a tendency to fail during extraction. In this series, it is likely that a combination of titanium alloy's fatigue properties secondary to notch sensitivity and osseointegration were responsible for this observation. The fatigue strength of titanium alloy is generally comparable to Stainless Steel 316L, but notch sensitivity in both commercially pure titanium and titanium alloy has been shown to significantly shorten the fatigue life of these implants in comparison to stainless steel [12–14]. 

**Figure 2:**
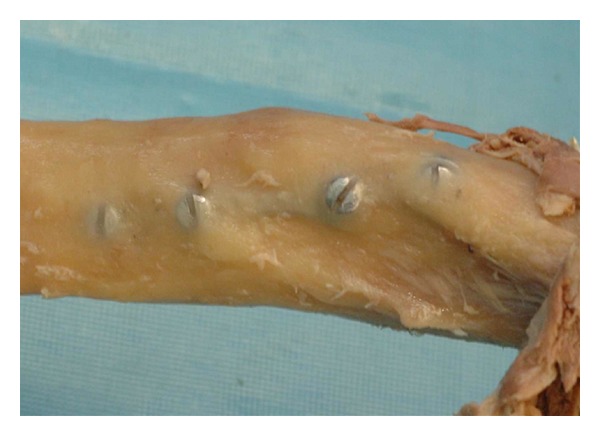
Bone ingrowth and on-growth onto metallic hardware in the humerus (cadaveric specimen). Due to a greater extent of osseointegration, greater removal torques will be necessary to extract the bone screws.

Osseointegration has been observed to occur within 3–10 months in titanium alloy [10, 11]. The degree of bone ingrowth and on-growth, however, continues to increase for years after initial implantation (Figure 2) [15]. Although there have been studies showing evidence of stainless steel osseointegration, it is generally accepted that commercially pure titanium and titanium alloy are more biocompatible and more likely to osseointegrate than stainless steel [16]. In our series, it is likely that as osseointegrataion became more complete, greater removal torques contributed to the failure of titanium alloy screws in this series [17]. Given these properties, we postulate that over longer periods and increased loading cycles, the development of micofractures and osseointegration contributed to screw breakage during implant removal.

One of the main limitations of this study was the small sample size. Secondly, the cases studied were not uniform; there were a wide variety fractures and hardware systems involved. In addition, due to the small number of cases it was necessary to include multiple surgeons. In addition, not all X-rays were available for review; therefore we were not able to account for the type of hardware, such as locking or non locking plate technology.

In this study, there appears to be a time-related association for bone screw failure during removal cases, and for titanium alloy in particular. This is likely due to the increased bone ingrowth and the adverse effect of notch sensitivity on titanium alloy's fatigue properties.

## Figures and Tables

**Table 1:** A summary of patient data.

Patient	Sex	Age (years)	Time from implantation until removal (months)	Implant Material	Location	Reason for removal	Broken Screws?
1	Female	48	11	Ti	PU	Nonunion	No
2	Female	27	23	Ti	DH	Symptoms	Yes
3	Female	34	5	Ti	PU	Infection	No
4	Male	40	48	SS	DH	Contracture	No
5	Male	30	74	SS	PU	Infection	No
6	Female	25	38	Ti	Both	Nonunion	No
7	Male	44	2	Ti	Both	Infection	No
8	Female	43	5	SS	PU	Symptoms	No
9	Female	30	12	Ti	PU	Symptoms	No
10	Female	57	9	Ti	PU	Symptoms	No
11	Female	46	33	SS	Both	Nonunion	No
12	Male	24	8	SS	PU	Infection	No
13	Male	47	4	Ti	DH	Infection	No
14	Female	40	9	SS	Both*	Symptoms	Yes
15	Male	17	53	Ti	Both*	Nonunion	Yes
16	Male	56	25	Ti	DH	Symptoms	Yes
17	Female	35	64	Ti	DH	Infection	Yes
18	Female	48	12	Ti	PU	Symptoms	No
19	Male	27	8	SS	DH	Infection	No
20	Male	66	16	Ti	PU	Nonunion	Yes
21	Male	28	7	Ti	Both	Nonunion	No

Ti: Titanium Alloy; SS: Stainless Steel; PU: Proximal Ulna; DH: Distal Humerus; *: Location of bone screw failure unknown.
